# Health Care Provider Adoption of eHealth: Systematic Literature Review

**DOI:** 10.2196/ijmr.2468

**Published:** 2013-04-16

**Authors:** Junhua Li, Amir Talaei-Khoei, Holly Seale, Pradeep Ray, C Raina MacIntyre

**Affiliations:** ^1^Asia-Pacific ubiquitous Healthcare research Centre (APuHC)The University of New South WalesSydneyAustralia; ^2^School of Public Health and Community MedicineFaculty of MedicineThe University of New South WalesSydneyAustralia; ^3^Discipline of InformaticsFaculty of Arts and BusinessUniversity of the Sunshine CoastSunshine CoastAustralia; ^4^National Centre for Immunization Research and Surveillance of Vaccine Preventable Diseases (NCIRS)SydneyAustralia

**Keywords:** technology acceptance, eHealth, health care provider, adoption

## Abstract

**Background:**

eHealth is an application of information and communication technologies across the whole range of functions that affect health. The benefits of eHealth (eg, improvement of health care operational efficiency and quality of patient care) have previously been documented in the literature. Health care providers (eg, medical doctors) are the key driving force in pushing eHealth initiatives. Without their acceptance and actual use, those eHealth benefits would be unlikely to be reaped.

**Objective:**

To identify and synthesize influential factors to health care providers’ acceptance of various eHealth systems.

**Methods:**

This systematic literature review was conducted in four steps. The first two steps facilitated the location and identification of relevant articles. The third step extracted key information from those articles including the studies’ characteristics and results. In the last step, identified factors were analyzed and grouped in accordance with the Unified Theory of Acceptance and Use of Technology (UTAUT).

**Results:**

This study included 93 papers that have studied health care providers’ acceptance of eHealth. From these papers, 40 factors were identified and grouped into 7 clusters: (1) health care provider characteristics, (2) medical practice characteristics, (3) voluntariness of use, (4) performance expectancy, (5) effort expectancy, (6) social influence, and (7) facilitating or inhibiting conditions.

**Conclusions:**

The grouping results demonstrated that the UTAUT model is useful for organizing the literature but has its limitations. Due to the complex contextual dynamics of health care settings, our work suggested that there would be potential to extend theories on information technology adoption, which is of great benefit to readers interested in learning more on the topic. Practically, these findings may help health care decision makers proactively introduce interventions to encourage acceptance of eHealth and may also assist health policy makers refine relevant policies to promote the eHealth innovation.

## Introduction

Poor health care outcomes lead to increased levels of morbidity and mortality, and obstruct countries’ prosperity and business profitability (eg, [1,2]). eHealth is an application of information and communication technologies (ICT) across health-related functions [3]. The benefits of eHealth, such as improved operational efficiency, higher quality of care, and positive return on investments have been well documented in the literature [4-6].

eHealth is an emerging field at the intersection of medical informatics, public health, and business, and refers to health services and information delivered or enhanced through the Internet and other related technologies [7,8]. Different eHealth applications have been used across countries, corresponding to their health needs and priorities. The World Health Organization (WHO) eHealth for Health Care Delivery (eHCD) program, for example, targeted primary health care in a number of countries in the Asia-Pacific region. Some of these countries have instigated telemedicine as a means of bringing specialist health care to rural communities, whereas some others have endeavoured to improve the safety and continuity of patient care through the use of electronic health records (EHR).

While there has been high interest in eHealth, the adoption and acceptance rates have not been high enough for health care systems to experience the maximal benefits eHealth has to offer [8]. Past experience of eHealth adoption in the United States, for example, informed us that the low adoption rate could be attributed to both macro-level factors (eg, supportive policies) from the perspective of the public, health care organization, and system, and micro-level barriers from the perspective of health care providers (eg, physicians’ perception about technological complexity, [9]).

A broad spectrum of research methodologies have been used to study eHealth adoption and acceptance factors based on information provided in published studies [9]. The methodologies include quantitative surveys [10], observations [11], qualitative focus groups [12], ethnographic studies [13], and personal intuition and experience [14]. According to the results of these studies, different eHealth adoption factors may have led to difficulty for decision makers to explicitly understand, measure, and decrease inhibiting factors or enhance facilitating forces [9]. Hence, there is a need to synthesize those insights and provide decision makers with a holistic view of eHealth adoption.

Health care providers are the key driving force in pushing eHealth initiatives [14]. eHealth implementation represents a disruptive change in the health care workplace. The change does not occur simply from the introduction of ICT infrastructure but may also require remodelling of the job design of interconnected health professionals to effectively and efficiently incorporate technology [15]. Without the presence of motivational forces (eg, health care providers’ dissatisfaction with the status quo), it is unlikely that the innovation process would be initiated. If health care providers resist change or do not possess attributes necessary for change (eg, adaptability and growth-orientation), the change process is less likely to proceed [16]. The objective of this paper was to identify and synthesize the factors influential to health care providers’ acceptance of various eHealth applications.

## Methods

### Overview

In light of the guidelines originally proposed by [17,18] and already applied in several systematic reviews (eg, [19]), we conducted a systematic literature review on eHealth adoption. For the specific objective of this study, the guidelines have been modified and 4 steps were taken: (1) identification of resources, (2) selection of relevant papers, (3) data extraction, and (4) data analysis and validation.

### Identification of Resources

A literature search was conducted between October and November 2011 using 8 online databases: Medline, Cinahl, Web of Science, PubMed, PsychInfo, ERIC, ProQuest Science Journals, and EMBASE. These databases were thought to be the most likely to publish eHealth adoption related work [20]. All search fields available from each search service were used. In each database, the search was repeated 3 times using the following phrases (operators came before keywords):  [“e-Health” AND “Adoption” OR “User Acceptance”] or [“eHealth” AND “Adoption” OR “User Acceptance”] or [“EMR” AND “Adoption” OR “User Acceptance”] or [“EHR" AND “Adoption” OR “User Acceptance”].

The terms “electronic medical records” (EMR) and EHR were separately used to search papers. This is because the EMR/EHR consists of patient health related information and forms the core of eHealth systems [8]. The inclusion of those papers increased the validity of the findings. Table 1 lists the number of papers found in each database using the search phrases. In summary, a total of 3315 papers were found, of which 420 papers were duplicated. The selection process excluded the repeated papers from the archive and produced a list of 2895 papers.

###  Selection of Relevant Articles

The full texts of the selected papers were reviewed for relevance. Papers with the following criteria were filtered out:

1. articles not written in English
2. articles that did not directly use the terms “adoption” and “eHealth” or related terms in the title, abstract, or entire text, with casual referencing of eHealth adoption related issues.
3. articles without empirical evidence
4. articles which discussed adoption or user acceptance of eHealth but not from the health care provider’s perspective

This examination process had two iterations. Finally, 93 relevant papers were selected.

### Data Extraction

The key information was extracted from the 93 papers. The extracted data included: (1) characteristics of the study (eg, year of publication and health care settings where the studies were conducted), (2) the study results and output—eHealth adoption factors. Relevant text was extracted or retyped verbatim and was added to a database.

**Table 1:** Identification of papers for review from 8 online databases.

Keywords	Medline	Cinahl	Web of Science	PubMed	PsycInfo	ERIC	ProQuest Science Journals	EMBASE (1980+)	Total	Duplicated results
User acceptance AND eHealth	2	3	15	2	2	1	73	2	100	-
User acceptance AND eHealth	6	0	7	8	3	0	45	2	71	20
User acceptance AND EMR	9	5	8	9	2	0	93	10	136	17
User acceptance AND EHR	13	2	15	12	3	0	57	10	112	20
Adoption AND eHealth	31	15	47	34	24	1	244	36	432	39
Adoption AND eHealth	29	9	29	44	28	1	155	30	325	74
Adoption AND EMR	89	30	67	97	12	3	395	101	794	87
Adoption AND EHR	165	83	106	187	17	1	607	179	1345	163
Total unrepeated articles retrieved									2895	-

### Data Analysis and Validation

**Figure 1:**
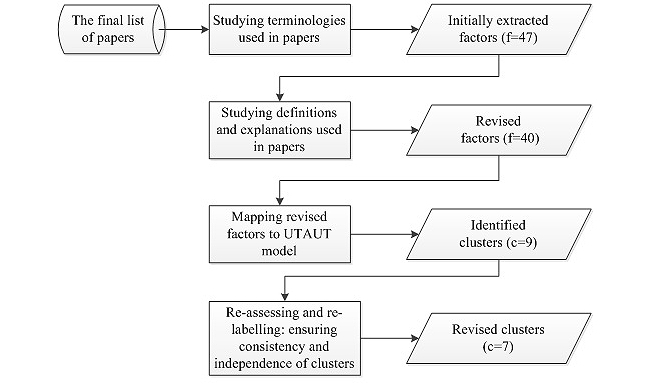
Data analysis process. f=number of factors; c=number of clusters.

Figure 1 illustrates the analysis process of the data collected in Step 3. Based on the terminologies or terms utilized in the papers, 49 eHealth adoption/acceptance factors were initially extracted. All citations used to identify the results were noted. The next activity was to study the definitions used in the papers. Factors with close relevance were combined, generating a list of 40 factors. For example, “time required to select, purchase, and install the eHealth system”, “time involved in learning to use the eHealth system and additionally required to become familiar with the system operation”, and “the degree to which use of the innovation is perceived as being time consuming” were all grouped to “time cost”.

Based on the perceived commonality of the themes, the 40 factors were analyzed and organized according to the Unified Theory of Acceptance and Use of Technology (UTAUT) by Venkatesh et al [21]. The UTAUT set out to integrate the fragmented theory and research on individual acceptance of information technology into a unified theoretical model, which highlights the importance of contextual analysis in developing strategies for technology implementation within organizations. This model accounts for 70% of the variance in usage intention—a substantial improvement over any of the original 8 models and their extensions. Within the UTAUT, 3 core constructs that impact on behavioral intention, and consequently use behavior, are *performance expectancy, effort expectancy,* and *social influence*, whereas the other core construct *facilitating conditions* has a direct impact upon use behavior. Four moderators (ie, *gender*, *age*, *voluntariness of use*, and *experience*) have also been incorporated in the UTAUT. Apart from the 4 core constructs and 4 moderators, another cluster of eHealth adoption factors, which could not be mapped against the UTAUT, was identified. Accordingly, the factors were initially grouped into 9 clusters (Figure 1).

To search for convergence among multiple sources of information and methods of data collection and analysis, a validity procedure was applied [22,23]. First, the eHealth adoption factors were reanalyzed within and across the clusters to ensure consistency and independence. The factors were regrouped into 7 clusters:

1. health care provider characteristics (eg, IT experience and knowledge, gender, age, and years in practice)
2. medical practice characteristics (eg, practice size and teaching status)
3. voluntariness of use
4. performance expectancy (eg, perceived usefulness and needs)
5. effort expectancy (eg, perceived ease of use)
6. social influence (eg, subjective norm)
7. facilitating or inhibiting conditions (eg, legal concerns)

The clusters were then given labels and reviewed once more for consistency. Reassessment and relabelling were performed for some papers. This step was repeated until a consensus was reached on the labels for clusters. In the final analysis, papers were reassigned to appropriate clusters. The resulting clusters represented another level of abstraction.

## Results

###  Characteristics of Selected Studies

This section presents the results of statistical analyzes on the characteristic data extracted from the 93 papers, including: (1) the growth of publications by years, (2) distribution by geographical areas, (3) types of research methodologies employed, (4) eHealth applications studied, (5) health care settings selected, and (6) study participants.

#### Growth of Publications

**Figure 2:**
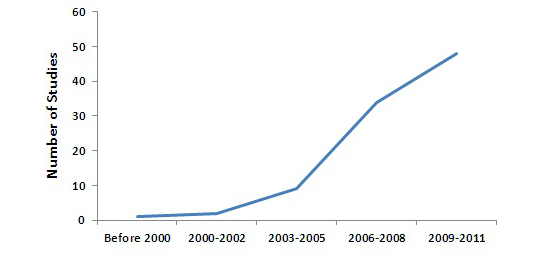
Growth of publications (based on our selected articles).

Figure 2 shows the growth in the publications. The growth represented by the curve was not linear, with a dramatic rise in the number of papers published after 2005.

#### Geographical Areas

The majority of the studies (72/93, 77%) were conducted in North America, followed by Europe (9/93, 10%), and Asia (7/93, 8%).

#### Research Methodologies

Quantitative methodology was predominately used by 57/93 studies. The number was nearly twice as large as that of qualitative studies.

#### eHealth Applications

The 93 papers addressed a wide range of eHealth applications. 57 targeted the EHR/EMR, which was defined as computerized medical information systems that collect, store, and display patient information [24]. Telemedicine/Telehealth was the second most popular application studied (addressed by 7/93 studies). Telemedicine frequently referred to the use of a wide array of technologies to deliver a range of medical services to persons at some distance from a health care provider [25]. The remnant studies examined the acceptance of other eHealth applications such as Intensive Care Information System (ICIS) [26], e-discharge which helps inpatient physicians to track pending tests at hospital discharge [27], Anesthesia Information Management System (AIMS) [28], and electronic logistics information system [29].

####  Health Care Settings

The majority of the studies were conducted in hospitals and office-based clinics (primary care). In some studies, multiple health care settings of different types were chosen to examine the eHealth acceptance issue. For example, Jha et al used survey data from stratified random sample of all medical practices in Massachusetts in 2005 to determine rates of EHR adoption and perceived barriers to adoption [30].

#### Study Participants

The majority of the studies (ie, 68/93) focused on physicians. Nurses and other health workers were recruited in 25 research projects on eHealth adoption and acceptance.

### eHealth Acceptance Factors

**Figure 3:**
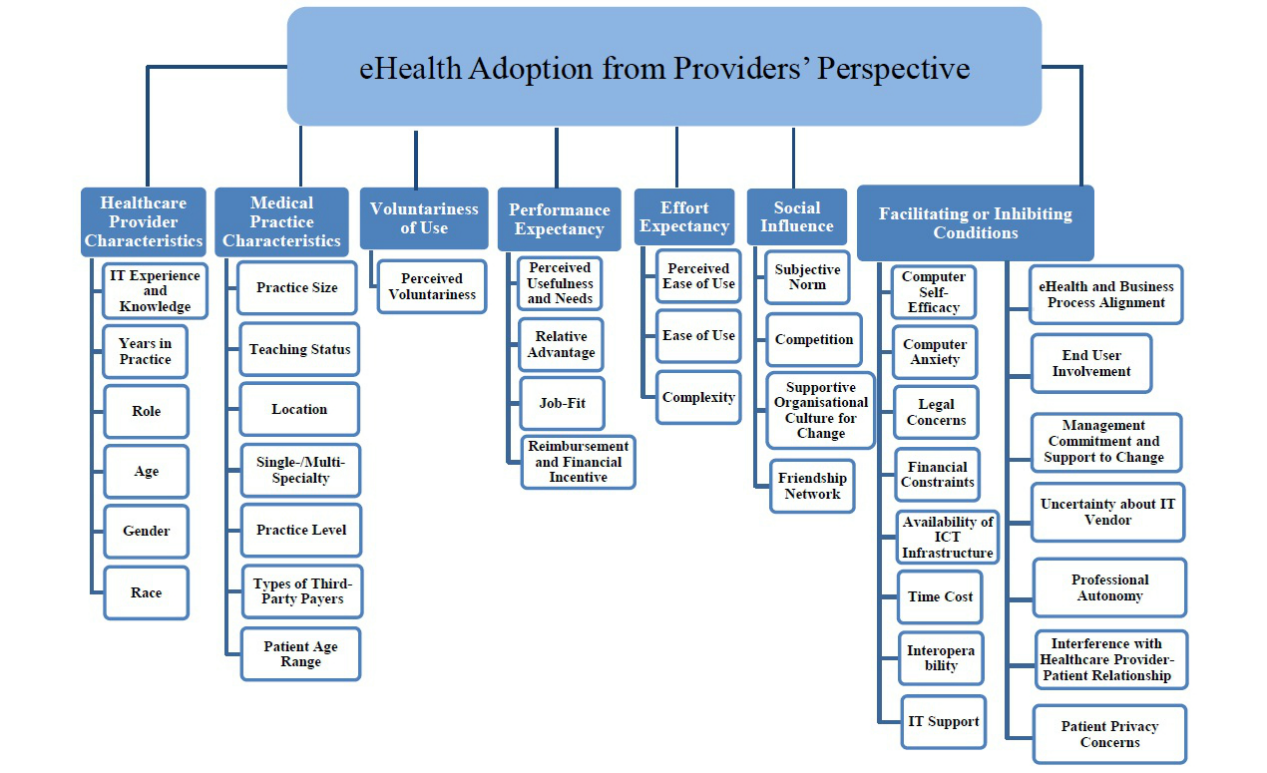
eHealth acceptance factors and clusters.

Through the data analysis and validation process, 40 factors were identified to be influential to the health care providers’ acceptance of eHealth and grouped into 7 clusters (Figure 3 and Table 2). A brief description of each cluster is provided below.

A health care provider’s characteristics included his/her information technology (IT) experience and knowledge, years in medical practice, professional role, age, gender, and race. Characteristics in relation to a health care provider’s medical practice included the practice size, teaching status, location, single or multi-specialty, practice level, types of third party payers, and patient age range. Voluntariness of use was defined as “the degree to which use of the innovation is perceived as being voluntary or of free will” [21]. Performance expectancy was defined as the degree to which a health care provider believes that using the eHealth system will help him or her to attain gains in job performance [21]. It included the perceived usefulness and needs, relative advantage, job-fit, and reimbursement and financial incentive. Effort expectancy was defined as the degree of ease associated with the use of the eHealth system [21]. It included perceived ease of use, ease of use, and complexity. Social influence was defined as the degree to which a health care provider perceives that important others believe he or she should use the new eHealth system [21]. It included the subjective norm, competition, supportive organizational culture for change, and friendship network. Facilitating or inhibiting conditions were defined as the degree to which a health care provider believes that an organizational and technical infrastructure exists to support use of the eHealth system [21]. It included the computer self-efficacy, computer anxiety, legal concerns, financial constraints, availability of ICT infrastructure, time cost, eHealth interoperability, IT support, eHealth and business process alignment, end user involvement, management commitment and support to change, uncertainty about IT vendor, professional autonomy, interference with the health care provider and patient relationship, and patient privacy concerns.

**Table 2:** eHealth acceptance factors under 7 clusters.

Cluster and factors		Definitions and citations
**Health care provider characteristics**		
	IT experience and knowledge	Generic IT skills (eg, typing skills) and experience [24,30-47] *Those who had little experience with computers were challenged by the process of learning how to use the computer in addition to learning the software* [43] Previous experience of computer use in medical practice or training in using particular eHealth systems [48-56] *Respondents with an electronic health record (EHR) were more likely to e-prescribe than those who did not have an EHR, and to have patients take a computer-generated prescription to the pharmacy* [55]
	Years in practice	Total years in practice since medical school graduation [32,48,57-61] *Based on the comments offered by those in practice for longer than 25 years in our study, it did not make sense to invest time or money at this point in their careers* [32]
	Role	Variation between physicians and other health professionals [53] *Physicians use most of the advanced features more than nonphysicians* [53] Variation between specialists and others [59,62,63] *high-end specialists, such as obstetrician-gynecologists, are less likely to be using EHR in their practice* [63]
	Age	Physical age [36,39,46,59,61,64-67] *EMR use was inversely associated with physician age* [65]
	Gender	Biological sex [39] *Females were less likely to use PDAs* [39]
	Race	A group of people of common ancestry, distinguished from others by physical characteristics [39] *African American and Hispanic physicians were more likely than Caucasian to indicate routine PDA use; Asian physicians reported using email with patients significantly less frequently than their Caucasian counterparts* [39]
**Medical practice characteristics**		
	Practice size	Number of physicians in the medical practice [36,39,48,57,58,60,61,65,67-72] *Physicians in practices with 11 or more physicians were most likely to use any EMR system, whereas physicians in solo practice were least likely to use EMRs* [65] Number of patient visits [24,32,61,72,73] *who saw fewer than ten patients per day, reviewed fewer than 20 medical records per day and handled fewer than ten calls daily, were statistically less likely to want to use a computer during a consultation; Those seeing fewer than ten patients daily were the most receptive to the use of handwriting* [32]
	Teaching status	Practices affiliated with academic institutions [58,70-72] *There was a statistically significant association between presence of students and residents in a practice and the practice’s use of an her* [71]
	Location	The medical practice in a rural setting or urban setting [40,61,68,72-74] *urban settings were significantly more likely to have adopted AIMS* [72]
	Single/Multi-specialty	Difference between those in a single-specialty practice and in a multi-specialty practice [39,65,66,68,75] *those in a multi-specialty group were more likely than those in a single specialty practice to routinely use EHRs* [39]
	Practice level	Distinctions between Primary, Secondary and Tertiary health care [36,58,60] *physicians whose practice consisted of a specialty other than primary care were more likely to use an EHR* [60]
	Types of third-party payers	Proportion of patients who are privately insured, Medicaid, Medicare, or uninsured [48,66,73,76] *Physicians with the highest percentage of Medicaid patients in their practices were significantly less likely to indicate using an EHR system when compared with those in the low-volume Medicaid group* [76]
	Patient Age Range	The age range of served patients’ [67] *doctors who treat HVE* ^a^ *were significantly less likely to adopt EHR* [67]
**Voluntariness of use**		
	Perceived voluntariness	The degree to which use of the innovation is perceived as being voluntary, or of free will [77] *Perceived voluntariness had a negative causality on behavioral intention to use telemedicine. These findings contradict those from prior IS literature that found a positive relation between voluntariness of use and intention to adopt* [77]
**Performance expectancy**		
	Perceived usefulness and needs	The degree to which a health care provider believes that using the eHealth system would enhance his or her clinical or non-clinical job performance [24,25,28,29,33,35,36,38,41,43,46,50,56,75,77-91] Perceived needs of adopting the eHealth system [42,79,92-94] *Participants from private hospitals or who owns a private practice reported that most of their patients are one-time customers and they do not expect them to come back. For private hospitals, about 30% of their patients are from out of the state (mostly from near towns and villages). Therefore, they do not keep their past medical records* [93]
	Relative advantage	The degree to which using an innovation is perceived as being better than using its precursor of practices [5,45,59-61,72,93,95,96] *physicians who used electronic prescribing were significantly more likely to view it as saving time than those who have not adopted the technology* [5]
	Job-fit	How the capabilities of the eHealth system enhance a health care provider’s clinical job performance [24,40,97] *no mechanism of alerting inpatient physicians that finalized test results were available for viewing (eg, by email or by an alert in the inpatient computer system* [97]
	Reimbursement and financial incentive	The degree of a health care provider’s perception of uncertainty over return on monetary investment [5,24,26,31,40,73,86,90,91,95,98] Availability of financial reward for a health care provider’s time investment in learning and using the eHealth system [36,54,70,86,92,99] *the availability of incentives for adoption of HIT were more likely to have EHRs than practices without such incentives* [70]
**Effort expectancy**		
	Perceived Ease of use	The degree to which a health care provider believes that using the eHealth system would be free of effort [5,25,28,29,38,40,46,47,52,54,56,68,74,75,81,84,87,88,90] *co-existence of paper and electronic records at the transition period, as an important barrier to EMR adoption* [74]
	Ease of use	The degree to which using the eHealth system is perceived as being difficult to use [5,27,28,35,41,45,46,52-54,64,77,84-86,89,91,97,100-103] *a perception that technical system deficiencies reduce the quality of clinical routines can result users’ resistance* [103] Location of ICT equipment for convenient use of the eHealth system [41,45,49,96,101,102] *Sometimes the physician practice does not have appropriate equipment to facilitate use of the e-Prescribing system as part of the existing workflow. For example, if they do not have a handheld device or computer in the examination room, the busy clinician needs to use a PC outside the examination room, adding an extra step to the workflow* [49]
	Complexity	The degree to which the eHealth system is perceived as relatively difficult to understand and use [24,26,35,37,45,46,54,79,84,86,89,93,96,100,101] *this study indicated that the EMR systems are very complex and difficult to learn, and this affects their attitude towards using the EMR systems* [93]
**Social influence**		
	Subjective norm	The health care provider’s perception that most people who are important to him or her thinks he or she should or should not adopt the eHealth system in question [40,59,77,91] *Patient resistance or not wanting their physicians to use EHR* [40]
	Competition	Perceived competitive advantage with eHealth [48,86,94] *adopt mobile technologies to gain a competitive advantage; adopting IS creates a competitive advantage by giving businesses new ways in which to outperform their rivals* [94]
	Supportive organizational culture for change	Leadership and presence of champions for the eHealth system adoption within a health care setting [24,35,38,43-45,74,79,86,96,104] *Health care professionals were likely to accept and participate in the process of eHealth adoption when the programs were introduced and promoted by a peer with considerable authority and influence and familiarity with the practices* [79] The degree of a health care provider’s perception of organizational culture (eg, learning culture) supportive to eHealth adoption [33,105] *The culture of the organization, including its supportive elements, influences both implementation and persistence of the work innovation* [33]
	Friendship network	Personal intimacy and interactions with personal friends [47] *Social influence affecting physician adoption of EHR was predominantly conveyed through interactions with personal friends rather than interactions in professional settings* [47]
**Facilitating or inhibiting conditions**		
	Computer self-efficacy	A health care provider’s self-judgment of his or her ability to use the eHealth system to accomplish clinical jobs or tasks [46,48,67,77,86]
	Computer anxiety	Evoking anxious or emotional reactions when it comes to adopting the eHealth system [24,33,40,77,80,92,106] *They are concerned that under certain circumstances, or as time passes, the systems will reach their limitations, become obsolete and will no longer be useful* [24]
	Legal concerns	The availability of the policy, regulation, and protocol supportive to using the eHealth system [31,54,74,78,79,82,93,95] *Regulation regarding sharing of clinical information between the various EMR users across settings of care could represent a complex issue. During interviews, some respondents expressed concern with respect to the application of the law related to patients’ consent in the context of EMR implementation* [74]
	Financial constraints	The degree of a health care provider’s perception of high monetary cost for adopting the eHealth system (ie, start-up costs and ongoing maintenance costs) and of the availability of financial resources to cover the cost [5,25,27,28,30-33,35,37,39,41,50,52,53,58,60,62,69,71-75,79,80,85-87,91,93,94,107-110] *respondents noted the lack of capital to invest in EHRs as an important or very important barrier to adoption* [73]
	Availability of ICT infrastructure	The degree of a health care provider’s perception of the availability of ICT infrastructure required for using the eHealth system [24,35,38,49,51,79,81,91,107]
	Time cost	Time required to select, purchase, and install the eHealth system [5,24,37,40,59,61,86,90] *Implementing an EMR means switching from paper-based to electronic based systems, and this involves transferring records between the two systems* [24] Time involved in learning to use the eHealth system and additionally required to become familiar with the system operation [25,28,31,32,37-39,41,44,46,50,53,55,57,60,62,71,72,74,85,87,91,92,109,110] *the time and effort involved in learning to use these technologies as a significant barrier* [31] The degree to which use of the innovation is perceived as being time consuming [24,35,84,86,90,93,97,99-101] *takes too much time to enter data in real time* [93]
	Interoperability	The degree of a health care provider’s perception of the ability of the eHealth system to exchange and use relevant clinical data within and across the health care setting [24,26,31,32,38,49,72,73,86,91,92,103,104] *Lack of ability to exchange clinical data with laboratories and hospitals is a major barrier for smaller physician practices* [31]
	IT support	The degree of a health care provider’s perception of the availability of experienced IT personnel for technical support (eg, troubleshooting emergent problems during actual usage of the eHealth system, and providing instructional and/or hand-on support to users before and during usage) [24,26,28,30,31,34-38,54,57,72,74,79,81,84,91,94,100] *the provision of good maintenance and user support systems greatly increases user acceptance of a new system* [84] The degree of a health care provider’s perception of the adequacy of training for the usage of the eHealth system [24,27,35,38,41,43,44,50,53,71,75,78,79,92,100,103,108] *This study found that inadequate training limits EMR utilization* [108]
	eHealth and business process alignment	The degree of a health care provider’s perception of the fitness of the eHealth system into the clinical workflow [29,32,77,96,97,99,103]
	End user involvement	The involvement of end users in the planning and implementation process of the eHealth system [24,38,75,83,84,86-88,103,104] *Clinicians’ resistance was also related to whether or not they had been involved in the design and implementation process* [103]
	Management commitment and support to change	The presence of management commitment and availability of management support for adoption of the eHealth system [24,33,45,75,79,81,82,87,88,91,92,103,109] *the implementers’ responses were supportive and addressed the issues related to the real object of resistance; the severity of resistance decreased* [109]
	Uncertainty about IT vendor	The degree of a health care provider’s perception of the availability of reputable and trustworthy external IT service providers in the market [24,29,49,52,106]
	Professional autonomy	The degree to which using the eHealth system is perceived by a health care provider as losing professional control over the conditions, processes, procedures, or content of his or her work according to the individual judgment in the application of his or her profession's body of knowledge and expertise [24,42,75,86-89,91,110,111] *With the implementation of EMRs, physicians are concerned about the loss of their control of patient information and working processes since these data will be shared with and assessed by others. Physicians’ perceptions of the threat to their professional autonomy are very important in their reaction to EMR adoption* [24]
	Interference with health care provider-patient relationship	The degree to which using the eHealth system is perceived as interfering the health care provider-patient relationship during their encounter [24,33,36,46,50,75,86-88,91,92,112] *physicians who value a close patient relationship have less positive attitudes about the EMR* [33]
	Patient privacy concerns	The degree of a health care provider’s perception of the security of patient information and protection of patient privacy [24,30,31,40,79,89,111,112]

^a^high volume of elderly

## Discussion

###  Comparative and Gap Analysis

Of the 93 papers, 57 examined the adoption/acceptance issue of EHR/EMR. EHR/EMR is a repository of health information in relation to a subject of care (ie, patient) in a computer processable form [113]. Li et al explained that electronic patient records form the core of any other eHealth applications and thus the success of these is very much dependent on the EHR/EMR adoption [114]. Although EHR/EMR can be utilized by all groups of health care providers (eg, physicians, nurses, and pharmacists), physicians were study participants among an overwhelmingly large number of publications.

After 2002-2004, there was a sharp increase in the number of publications. A majority of these studies were conducted in the United States. According to Burt et al [115], EHR adoption in the United States was significantly low until 2005, with less than 18% of physicians used EHR at their office. After 2005, there was a great increase in EHR adoption levels across the United States [115], making more health care settings available for eHealth acceptance research.

Most of the 93 studies used a quantitative research methodology to measure eHealth adoption/acceptance variables and test hypotheses. A small percentage applied models or theories on individual acceptance of information technology (eg, Technology Acceptance Model, TAM [116-118]). The results supported the models in predicting the adoption behavior in the health care context. The most applied model was the TAM, which proposed a method of evaluating user acceptance through his/her beliefs, attitudes, intentions, and actual technology adoption behavior. Within these studies [25,29,41,42,75,77,79,81,83-85,87,88,102], the factors influential to health care providers’ acceptance of eHealth included their perceived usefulness and needs, perceived ease of use, and all of the facilitating or inhibiting conditions.

Few studies (eg, [41]) have successfully tested the applicability of the UTAUT model by Venkatesh et al [21]. Using the definition of the UTAUT constructs, we analyzed and organized the eHealth acceptance factors that we found. The mapping work demonstrated that the UTAUT model is a useful framework for applying and organizing literature, which is of great benefit to readers interested in learning more on the topic [119]. Nevertheless, it was found that half of the health care provider characteristics (years in practice, role, and race) as well as medical practice characteristics identified from this literature review have not yet been covered in the UTAUT. Further, some studies also showed significant correlations among the identified factors. Perceived usefulness had the strongest impact on health care providers’ behavior intention [88], whereas their perceived usefulness was influenced by the perceived ease of use, eHealth and business process alignment, end user involvement, management commitment and support to change, health care provider-patient relationship, and IT experience and knowledge [25,28,33,56,77,83,86-88]. The variance of the perceived ease of use was associated with the computer self-efficacy, end user involvement, management commitment and support to change, as well as health care provider-patient relationship [77,88]. These correlations have not been incorporated in the UTAUT. Our efforts to map eHealth acceptance research results against the UTAUT model suggested that health care settings could potentially extend theories on information technology adoption due to their complex contextual dynamics.

In some of the papers, significant correlations were not necessarily found between acceptance factors on the list (particularly those of individual characteristics and medical practice characteristics) and health care providers’ usage intention or actual use of eHealth. Chavis’s study [105], for example, did not demonstrate a significant positive correlation between individual characteristics (ie, job role and age) and technology adoption. This result can be explained with the UTAUT model: the age acts as a moderator rather than a factor directly impacting upon the behavioral intention or use behavior. Russell et al found that health care providers in large practices were not more likely to use an EMR [112]. Others [24,40,57,69,120] argued against that, suggesting that larger practices tended to “have access to the potentially greater resources” (financial and human resources) required for the eHealth system delivery and adoption, and have extensive internal IT assistance and training.

Apart from the contradicting findings among these studies, some acceptance factors can also be context sensitive. Given that most of the 93 studies were conducted in the United States, the types of third-party payers (which is by definition the proportion of patients who are privately insured, Medicaid, Medicare, or uninsured), for example, reflects the health insurance scheme specifically in the United States context. In the future, further studies particularly in health care settings of other countries, are required in order to improve the understanding of eHealth adoption phenomenon in a global context, as well as to extend the theory and research on individual acceptance of information technology.

### Limitations

Here are a few major limitations of this literature review. Although efforts were made to include all research papers on health care providers’ acceptance of various eHealth applications, some may not have been identified due to selected search phrases. In order to at least include those papers, which can help us increase the validity of the findings, the supplementary search keywords “EHR” and “EMR” were both used as previously discussed.

The review was limited also due to the selection of the databases. Although they are the outlets that were deemed most likely to publish eHealth acceptance-related work, some papers may have been missed. We tried to compensate for this potential loss by ensuring that all selected databases were searched to their full extent.

Mapping the identified eHealth adoption factors against the UTAUT model can be subjective. We attempted to maximize the accuracy and appropriateness of our mapping work by applying the validity procedure.

### Practical Implications

####  To Decision Makers at Health Care Settings

The study results could help decision makers at the health care setting systematically understand facilitating forces and inhibiting factors influential to the health care providers’ acceptance of eHealth, and thus proactively introduce interventions for the adoption success. For example, health care providers may lack the adequate computer skills to use eHealth systems or had previous negative technology experiences [49,121]. IT support before, during, and after initial eHealth implementation can provide a smooth transition to their reengineered job routine and overcome their technology phobia, hence facilitating eHealth acceptance and use (eg, [27,78,81]). IT support includes, but is not limited to training, provision of guideline documents, and troubleshooting [50,123,124].

Training can take various forms such as group training or one-on-one training, which is ideal in all circumstances [122]. One-on-one training needs to set expectations, teach health care providers about the eHealth system features, customize the technology for each particular specialty, and help them to integrate the system (eg, e-Prescribing) into their medical practice workflow [49].

Guideline documents as a knowledge source promote authentic translation of domain knowledge and reduce the overall complexity of the implementation task [123]. Each care provider should be provided with a manual containing step-by-step instructions for the system’s use [124].

Real time troubleshooting (especially through internal resources) facilitates the effective use of the eHealth system and becomes essential to the system success in terms of actual usage [49,124]. Health care providers need to know how to access it when required [124]. A feedback mechanism (eg, online help) allows health care providers to document a problem that they are having with the system and then to receive prompt feedback [13,125]. Compared with external support services from the IT vendor, internal IT staff is more familiar with the work environment and related needs, and may respond more quickly to an urgent request [124].

Another example is eHealth/business process alignment. Workflow is associated with routine processes, characterized by a fixed definition of tasks and an order of execution [126]. The eHealth system needs to be designed in close collaboration with health care providers so that it truly assists their medical practice [122,127,128]. The collaboration between IT vendors and clinical sites is to understand the site's workflow and determine the most suitable IT solution [124,129]. After the workflow is analyzed thoroughly with health care providers’ involvement, their participatory process is also essential to fine-tune the system’s capabilities [128]. Extensive software testing of the vendor's claims for the baseline functionality and system adaptability to local needs is critical before the implementation, as health care providers' frustration from software problems can promptly escalate and result in resistance to continue using the system [128].

#### To Policy Makers at the Health Sector

By synthesizing the evidence from the literature, our study may also assist policy makers at the health sector in refining or developing relevant policies to push eHealth innovation. eHealth adoption and ongoing maintenance requires a large capital investment [131-133]. While the government in some cases funds the start-up cost of an eHealth project (eg, the EMRX system in Singapore), health care providers may still need to undertake the operation and enhancement cost of their system [8]. In small or independent medical practices, there is lack or absence of internal capacity for system maintenance; eHealth vendors alternatively provide all these services but often charge high fees. Due to financial constraints, system maintenance represents a vulnerable spot for the entire effort of eHealth and many practices underperform [130]. To address this challenge, the development of programs such as zero-interest or revolving loans that make capital available to health care provider groups at low interest rates is essential, particularly in small or independent practices [48,106,130].

Another important issue is interoperability. Bates commented that the interoperability between eHealth applications and seamless and reliable clinical information exchange is a key to making EHR use a cornerstone of practice [130]. Even if physicians started to use an EHR system, they might still be unable to seamlessly share some other patient information (such as laboratory and radiology results stored in Laboratory Information Systems, LIS, and Picture Archiving and Communication Systems, PACS) for clinical decisions [130]. According to a recent analysis, $77.8 billion USD could be saved annually by interoperable clinical information exchange among key stakeholders in the health care delivery system [131]. The government should take stronger position to create a database of eHealth vendors whose products meet certain standards and enable clinical information exchange and to certify these products [31,82]. The certification effort would also minimize health care providers’ uncertainty over the selection of a viable and sustainable product from hundreds of IT vendors in the market [68,106].

Legal and regulatory changes can be required to address eHealth adoption related issues [130,132]. For example, the Medicines Regulations (1984) and the Misuse of Drugs Regulations (1977) in New Zealand, which governs respectively the form of medication prescriptions and controlled substances, stated that indelible text and practitioners’ handwritten signature was required for a legitimate prescription. To facilitate the adoption of electronic prescribing and dispensing of medicines, the Health Department of Commonwealth has amended the National Health (Pharmaceutical Benefits) Regulations [8]. These amendments came into effect from March 1, 2007 and the electronic prescribing and dispensing process has been additional and separate to the already existing paper-based process. The states and territories have continuously been taking steps to remove any legal barriers to the adoption of the electronic process in each jurisdiction.

### Concluding Remarks

In this 4-step literature review, 40 factors were identified to be influential to health care providers’ acceptance of eHealth and organized in accordance with the UTAUT model. The findings may help decision makers at health care settings and policy makers at the health sector to better understand eHealth adoption issues and take action to facilitate the eHealth innovation process. Our work also suggests further studies to extend theories on information technology adoption.

## Abbreviations

AIMS: Anesthesia Information Management System
eHCD: eHealth for Health Care Delivery
EHR: electronic health records
EMR: electronic medical records
HVE: high volume of elderly
ICIS: Intensive Care Information System
ICT: information and communication technologies
IT: information technology
LIS: Laboratory Information Systems
PACS: Picture Archiving and Communication systems
TAM: Technology Acceptance Model
UTAUT: Unified Theory of Acceptance and Use of Technology
WHO: World Health Organization
